# *Mycoplasma hominis* and *Candidatus* Mycoplasma girerdii in *Trichomonas vaginalis*: Peaceful Cohabitants or Contentious Roommates?

**DOI:** 10.3390/pathogens12091083

**Published:** 2023-08-25

**Authors:** Valentina Margarita, Antonella Congiargiu, Nicia Diaz, Pier Luigi Fiori, Paola Rappelli

**Affiliations:** 1 Department of Biomedical Sciences, University of Sassari, Viale San Pietro 43/B, 07100 Sassari, Italy; vmargarita@uniss.it (V.M.); a.congiargiu@phd.uniss.it (A.C.); ndiaz@uniss.it (N.D.); fioripl@uniss.it (P.L.F.); 2 Mediterranean Centre for Disease Control (MCDC), 07110 Sassari, Italy; 3 Microbiology Unit, University Hospital of Sassari (AOU), 07110 Sassari, Italy

**Keywords:** *Trichomonas vaginalis*, *Mycoplasma hominis*, *Candidatus* Mycoplasma girerdii, trichomoniasis, symbiosis, endosymbiont, vaginal infection

## Abstract

*Trichomonas vaginalis* is a pathogenic protozoan diffused worldwide capable of infecting the urogenital tract in humans, causing trichomoniasis. One of its most intriguing aspects is the ability to establish a close relationship with endosymbiotic microorganisms: the unique association of *T. vaginalis* with the bacterium *Mycoplasma hominis* represents, to date, the only example of an endosymbiosis involving two true human pathogens. Since its discovery, several aspects of the symbiosis between *T. vaginalis* and *M. hominis* have been characterized, demonstrating that the presence of the intracellular guest strongly influences the pathogenic characteristics of the protozoon, making it more aggressive towards host cells and capable of stimulating a stronger proinflammatory response. The recent description of a further symbiont of the protozoon, the newly discovered non-cultivable mycoplasma *Candidatus* Mycoplasma girerdii, makes the picture even more complex. This review provides an overview of the main aspects of this complex microbial consortium, with particular emphasis on its effect on protozoan pathobiology and on the interplays among the symbionts.

## 1. Introduction

*Trichomonas vaginalis* is an obligate parasite that colonizes the human genital tract causing trichomoniasis, the most common nonviral sexually transmitted infection. The World Health Organization estimated that there are more than 150 million new cases per year, with a global prevalence of 5.3% for women and 0.6% for men aged 15–49 years in 2016 [1].

*T. vaginalis* is a flagellated protist that is unable to survive in the external environment and has humans as the only natural host. The protozoon presents as pear-shaped trophozoite measuring 10 µm × 7 µm on average. It lacks a true cystic stage, even though in undesired conditions it can transform into pseudocysts [2]. Four anterior flagella and a fifth one incorporated in the free margin of an undulating membrane confer the characteristic motility to *T. vaginalis*. Its peculiarity is the presence of hydrogenosomes, metabolic organelles that share a common ancestor with mitochondria, involved in the parasite’s metabolic pathways. Hydrogenosomes are implicated in the fermentation of carbohydrates, which represent the main energetic source for the protozoon, under both aerobic and anaerobic conditions [3]. The sequencing of the *T. vaginalis* genome in 2007 highlighted that there are ~60,000 protein-coding genes organized into six chromosomes, with 65% of its content consisting of repetitive sequences, including transposable elements [4].

The infection in women affected by trichomoniasis ranges from asymptomatic (~50% of cases) to severe vaginitis. Symptoms, when present, include yellowish-green vaginal discharge, vulva itching, dysuria, abdominal pain and, less frequently, a strawberry cervix. In women, trichomoniasis is associated with important sequalae including pelvic inflammatory disease, increased risk of HIV acquisition and shedding [5], increased risk of HPV infection and invasive cervical cancer [6,7]. *T. vaginalis* infection is also associated with infertility and adverse pregnancy outcomes, such as preterm birth, premature membrane rupture and low birth weight [8]. In men, the protozoon infects urethra and prostate, in most cases without causing any symptoms. However, its presence is correlated with infertility and an increased risk of prostate cancer [9,10].

The worldwide distribution and prevalence of trichomoniasis is certainly underestimated due to the high number of asymptomatic infections and to the limited sensitivity of the most commonly used diagnostic methods. In fact, diagnosis is most frequently carried out by microscopic examination of a “wet mount” from vaginal swabs. The method is fast and cost effective, but shows a sensitivity of around 60% [11]. Culture of *T. vaginalis* from clinical samples has long been considered the gold standard for the diagnosis of infection, thanks to its higher sensitivity when compared to microscopy. However, this method has several disadvantages, including the need for an equipped laboratory and the long times required to obtain a diagnosis. As a result, molecular methods, which show the highest sensitivity and specificity for the detection of *T. vaginalis*, are increasingly used in diagnostic laboratories. Nucleic acid amplification tests (NAATs) are mostly RT-PCR-based syndromic panels that allow for the simultaneous detection of several sexually transmitted diseases, and are now widely used in diagnostic laboratories [12].

Host epithelial cells damage and alteration of resident microbiota are the two main mechanisms that *T*. *vaginalis* put in place to exert its pathogenicity. The protozoon is an extracellular organism, and adhesion to host epithelial cells is a prerequisite to create a stable infection and to damage host tissues. Upon contact with the host mucosa, *T. vaginalis* transforms from pear-shaped to amoeboid, thus increasing its adhering surface to vaginal epithelial cells. Adhesion to target cells is followed by the secretion of molecules with cytotoxic activity, such as proteases and pore-forming proteins, that are responsible for the epithelial damage observed during infection [13].

The presence of the protozoon can also profoundly influence the composition of the vaginal microbiota. Ravel et al. described five types of vaginal microbiota in women in childbearing age, the so-called community state types (CSTs), composed of aerobic and anaerobic microorganisms that establish dynamic interactions among each other and with the host. Four of them are characterized by the predominant presence of bacteria of the genus *Lactobacillus*, which exerts a protective effect on the host by lowering the vaginal pH and by producing antimicrobial compounds that create an inhospitable environment for potential pathogens [14]. By contrast, CST-IV has no specific dominant species and presents a greater proportion of anaerobic bacteria compared with other CSTs, including pathogenic bacteria associated to bacterial vaginosis [15]. Interestingly, 72% of women with trichomoniasis present with CST-IV type microbiota. In fact, *T. vaginalis* is, in most cases, associated with vaginal dysbiosis, outlining a significative interplay of the protozoon with vaginal microbiota, confirmed by the demonstration that bacteria of the dysbiotic microbiota can enhance protozoan pathogenicity [15]. Moreover, the protozoan is able to establish true symbiotic relationships with specific components of the vaginal microbiota [16,17].

In 1985, Wang et al. described the presence of dsRNA viruses within trichomonad cells. Since then, four different *Trichomonas vaginalis* dsRNA virus species have been identified within the protozoon [18,19]. *T. vaginalis* are small (4.5–5kbp), nonfragmented dsRNA viruses belonging to *Trichomonasvirus* genus of the Totiviridae family. Interestingly, the four different virus species can coexist in the same *T. vaginalis* cell [20]. Fraga and colleagues have observed that the presence of *T. vaginalis* increases in vitro the adherence to human cells of *T. vaginalis* isolates and influences the severity of symptoms in patients affected by trichomoniasis [21]. It is conceivable that the presence of *T. vaginalis* within the parasite may result in upregulation of the virulence genes of the protozoan affecting the severity of trichomoniasis symptoms and the virulence of the parasite [22].

In 1975, apparently intact Mollicutes were observed using electron microscopy within the cytoplasm of *T. vaginalis* cells [23], but only in 1998 were they isolated and identified as *Mycoplasma hominis* [24]. Since then, it has been demonstrated that an actual endosymbiosis is established between the two microorganisms. The intracellular location of the bacterium in *T. vaginalis* has been demonstrated using gentamicin protection assays and confirmed using confocal and electron microscopy [25,26].

*M. hominis* is a bacterium belonging to the class *Mollicutes* and is an obligate parasite of the human urogenital tract. It is the simplest self-replicating microorganism known, with one of the smallest genomes described so far [27]. *M. hominis* is characterized by the absence of a rigid cell wall and is therefore innately resistant to β-lactams and to all antibiotics that target the cell wall. The very small genome reflects on the reduced metabolic abilities of the bacterium and makes it strongly dependent on host cell metabolism. *M. hominis* is found as a commensal of the genitourinary tract of healthy individuals but is also associated with a wide range of diseases, such as pelvic inflammatory disease, cervicitis and pyelonephritis [28]. Colonization rates greatly vary worldwide, ranging from 1.3 to 51% [29]. It has been demonstrated that *M. hominis* is associated with alterations in the vaginal flora, including bacterial vaginosis. A recent study of Rumyantseva et al. demonstrated a threefold increase in *M. hominis* prevalence in reproductive-aged women with bacterial vaginosis compared to healthy women [30].

The symbiosis between *T. vaginalis* and *M. hominis* is the only one described so far involving two obligated human parasites capable of inducing independent diseases in the same anatomical site.

Rather surprisingly, in 2013, a further microorganism was observed in association with *T. vaginalis.* Martin and colleagues described the 16S rRNA sequence of a new *Mycoplasma* almost exclusively in vaginal secretions of women with trichomoniasis, and named the bacterium Mnola [31]. Shortly thereafter, Fettweis et al. characterized the new Mycoplasma using metagenomic strategies, and renamed it as *Candidatus* Mycoplasma girerdii, in honor of the American gynecologist P.H. Girerd. Phylogenetic analyses confirmed that *Ca.* M. girerdii belongs to the genus *Mycoplasma*, with 94% of sequence identity with an uncultivated *Mycoplasma* found in veterinarian specimens, 85% similar to the closest human pathogen, *Mycoplasma genitalium*, and only 78% similar to *Mycoplasma hominis*. *Ca.* M. girerdii possesses a small genome (~619 kb), which accounts for the strict metabolic dependence of the bacterium [32].

Attempts to axenically cultivate *Ca.* M. girerdii in vitro have been unsuccessful so far. Biological features of the bacterium have been therefore inferred only through metagenomic analysis until the recent work of Margarita et al., in which an in vitro model of cocultivation of *Ca.* M. girerdii with *T. vaginalis* was developed [33]. In fact, metabolic in silico reconstructions suggest that *Ca.* M. girerdii is glycolytic, encoding all enzymes needed to use glucose as an energy source and lacks gluconeogenesis, Krebs cycle, and enzymes for purine, pyrimidine, and amino acid synthesis. These data were supported by in vitro RNA-Seq analyses describing a high number of mapped reads involving various putative amino acid transporters, enzymes involved in amino acid catabolism and fully annotated glycolytic pathways, suggesting energy generation via these pathways [33]. These features reflect a limited metabolic capability, typical of *Mollicutes*, making *Ca.* M. girerdii strongly dependent on the host, which provides a protected niche and nutrients necessary for survival [34].

Data obtained so far support the hypothesis that *Ca* M. girerdii is a strict endosymbiont of *T. vaginalis*, thus adding a new member to the already populous family of microorganisms that live in close association with the protozoan.

In this review, we describe the relationships between *T. vaginalis* and the two mycoplasmas and the impacts of the presence of the symbionts on the protozoan pathobiology. In particular, we focus on the interactions that occur between the two symbiotic bacteria when cohabiting in the same *T. vaginalis* cell.

## 2. *T. vaginalis* and Its Endosymbionts: *M. hominis* and *Ca.* M. girerdii

Since its first description by Rappelli et al. [24], the strict association between *T. vaginalis* and *M. hominis* has been largely confirmed both using PCR in protozoan strains isolated in different geographical regions and through epidemiological studies, with an association rate ranging from 5% to 89% [24,35,36,37,38]. On the contrary, given the recent discovery, epidemiological data on the presence of *Ca.* M. girerdii in the vaginal microenvironment are extremely limited.

Fettweis and colleagues, analyzing 16S rRNA gene-based microbiome profiles of patients as part of the Vaginal Human Microbiome Project [32], observed DNA specific for *Ca.* M. girerdii in 36 out of 63 vaginal samples of women with trichomoniasis, while its presence in healthy women was extremely rare. The presence of *Ca.* M. girerdii in vaginal samples has also been investigated in women diagnosed with trichomoniasis in the USA, China and Austria, with percentages of 63%, 42% and 20%, respectively [31,39,40], demonstrating that the association between *T. vaginalis* and the symbiont *Ca.* M. girerdii, as well as in the case of *M. hominis*, is distributed worldwide. In a high percentage of cases, the same clinical samples with *Ca.* M. girerdii were also positive for *M. hominis.* To demonstrate that the contemporary presence of the two Mycoplasmas was due to a real multiple symbiosis and not merely to a coinfection, *T. vaginalis* isolates from long-term in vitro cultures were analyzed using real-time PCR, showing that *Ca.* M. girerdii-specific DNA was present in 46 out of 75 *T. vaginalis* Italian isolates analyzed. Among them, only four were infected by *Ca.* M. girerdii alone, while 42 presented also *M. hominis* [33]. The double infection in the same trichomonad strain was recently confirmed by Xu et al. [39] (Table 1).

**Figure 1:**
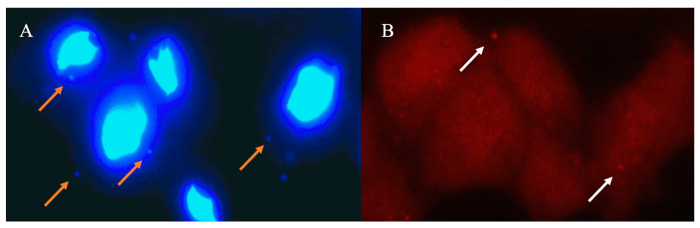
*T. vaginalis* cells coinfected by *M. hominis* and *Ca.* M. girerdii stained with DAPI (**A**) and anti-*M. hominis* antibody (**B**). The two images were captured on the same plan using different filters. In A, both symbionts are highlighted, while in B, solely *M. hominis* are decorated using specific rhodamine-conjugated antibodies. Orange arrows indicate *Ca.* M. girerdii and white arrows indicate *M. hominis*.

The demonstration that the two mycoplasmas can cohabit in the same trichomonad cell was obtained in immunofluorescence by Margarita et al. [33] by combining DAPI and specific anti-*M. hominis* antibodies (Figure 1). In Figure 1a, both mycoplasmas are highlighted in the trichomonad cytoplasm using DAPI staining, while in Figure 1b, specific antibodies decorate solely *M. hominis*. Lacking mitochondria, the trichomonad cell is free of cytoplasmatic DNA, so DAPI staining shows only the symbiotic mycoplasmas in the cytoplasm.

By using an in vitro model system, Margarita et al. showed that *Ca.* M. girerdii can live both on the surface and in the intracellular compartment of *T. vaginalis*, and that the replication of bacteria occurs mainly intracellularly. Nevertheless, an intracellular competition between *Ca.* M. girerdii and *M. hominis* may occur when in symbiosis with *T. vaginalis.* In fact, the capability of *Ca.* M. girerdii to establish a stable infection within the protist decreases when it is already infected by *M. hominis*, at least in vitro [33]. These results suggest that *T. vaginalis* might be more susceptible to infection by *M. hominis* than that by *Ca.* M. girerdii, or that the presence of *M. hominis* could render the protozoon less susceptible to be colonized by *Ca.* M. girerdii.

The high frequency of the association among *T. vaginalis* and the two Mycoplasmas supports the thesis that all three microbial species may obtain benefits when they are together.

Mutual benefits of the symbiosis between *T. vaginalis* and *M. hominis* have been extensively described in recent years, but the advantages for the protozoon deriving from the association with *Ca.* M. girerdii have yet to be defined. By comparing the growth kinetics of *T. vaginalis* alone and associated with one or both bacteria, Margarita and colleagues showed that both *M. hominis* and *Ca.* M. girerdii, in single or double infections, promoted the parasite growth rate [33,41]. *Ca.* M. girerdii, such as *M. hominis*, may influence trichomonad multiplication rate upregulating central metabolism with a shift from hydrogenosomal to cytosolic lactate and malate fermentation, along with an increase in amino acid catabolism.

## 3. Influence of *Ca.* Mycoplasma girerdii and *Mycoplasma hominis* on the Pathobiology of *T. vaginalis*

*T. vaginalis* is an extracellular pathogen that needs to strictly adhere to human epithelial cells to establish an infection and to exert its cytopathic effect [42]. Thanks to its capability to transform from pear-shaped to ameboid upon contact with the urogenital epithelium of men and women, *T. vaginalis* increases the surface of contact with the target membranes creating a strict cell-to-cell association, that is a prerequisite for its cytopathic effect. *T. vaginalis* adhesion is a complex process in which several parasite factors are involved. A number of surface proteins potentially involved in trichomonad adherence to the host have been described, yet their effective role has in most cases yet to be defined. *T. vaginalis* surface is characterized by the presence of lipoglycan (TvLG), which has been demonstrated to be involved in adhesion and cytotoxicity of parasites to target cells. In fact, TvLG binds to host cell galectin-1 [43] and -3 [44], playing a role in the first phase of infection [13].

Upon adhesion to target cells, the protozoon secretes several virulence factors, such as proteases and pore-forming proteins, that play an important role in host epithelial damage. Although a number of proteases and saposin-like proteins showing pore-forming activity implicated in protozoan pathogenesis have been identified, much of the host–parasite interaction remains to be unraveled [13].

It has now become clear that the pathobiology of *Trichomonas vaginalis* involves multiple interactions not only with host tissues and immune response, but also with its symbionts. In recent years, several studies have shown the important role of *M. hominis* in influencing the ability of *T. vaginalis* to adhere and induce a cellular damage to epithelial cell. The presence of *M. hominis* was associated with an exacerbation of *T. vaginalis* pathogenicity by Vancini and colleagues, who observed how trichomonad strains naturally infected by *M. hominis* led to more pronounced cellular damage of vaginal epithelial cells (VECs) in vitro, compared to isolates devoid of the symbiont. They also demonstrated that *T. vaginalis* harboring *M. hominis* showed an increased ameboid transformation rate and phagocytic activity [45].

Several studies highlighted a strong variability among *T. vaginalis* strains in their pathogenic features [42]. In order to overcome this strain-to-strain phenotypic variability, isogenic *T. vaginalis* with and without *M. hominis* have been used in several studies to investigate on the possible effects of the symbiont on protozoan features. To create isogenic pairs, *T. vaginalis* natively harboring *M. hominis* were either cured from the bacterial infection or, conversely, protozoa originally *Mycoplasma*-free were stably infected in vitro with the bacteria [46,47].

By using isogenic strains, it has been shown that the ability of *T. vaginalis* to adhere to human epithelial cells is ~10-fold enhanced in protozoa symbiotically associated with one or both *Mycoplasma* species. These findings are supported by RNAseq data showing that 8 out of 11 TvBspA proteins found in highly adherent *T. vaginalis* strains, potentially implicated in the physical interaction of protozoa with other cells [48], were upregulated when infected by *M. hominis* and/or *Ca.* M. girerdii. These data support the idea that *Ca.* M. girerdii, such as *M. hominis*, can increase the capability of protist to adhere to host cells.

Also, *Ca.* M. girerdii, like *M. hominis*, seems to enhance the protozoan cytolytic activity. In 2016, Margarita et al. demonstrated that hemolytic activity of the reference *T. vaginalis* G3 strain is more than doubled when symbiotically associated with *M. hominis* [41]. More recently, the same approach was used to investigate on the effect of the presence of *Ca.* M. girerdii on the virulence of the parasite. *T. vaginalis* G3 strain experimentally infected by *Ca.* M. girerdii, alone or in association with *M. hominis*, showed higher hemolytic activities than the same isogenic mycoplasma-free strain. These results were confirmed and supported by RNAseq analysis: 7 of the 11 transcribed saposin-like proteins(TvSaplip) genes that potentially mediate the pore-forming activity underlying hemolysis [13] were significantly upregulated in presence of *Ca.* M. girerdii and/or *M. hominis* [33].

Pathogenesis of trichomoniasis is the result of both parasite factors and host immune response. *T. vaginalis* infection, whether it is symptomatic or asymptomatic, is usually accompanied by leukocyte infiltration, as well as by high levels of secreted proinflammatory cytokines. In fact, *T. vaginalis* takes advantage of a strong inflammatory immune response to generate tissue damage and to develop infection [49,50].

The presence of *M. hominis* in trichomonad cells seems to influence even this pathogenic aspect. In 2013, Fiori’s group investigated the role of *M. hominis* in *T. vaginalis* over host innate immunity, showing that the symbiont synergistically upregulates IL-8, IL-1β, and TNF-α production by the human monocytic cell line THP-1. Moreover, THP-1 cells secreted IL-23, a Th17-polarizing cytokine, upon contact with *T. vaginalis* harboring *M. hominis* but not with *T. vaginalis* alone [51].

Mercer and colleagues compared the capability of killing human primary B-cells, T-cells, and monocytes of an isogenic *Mycoplasma*-free *T. vaginalis* strain versus a naturally *Mycoplasma*-infected strain. Results showed that the presence of *M. hominis* induced a qualitative and quantitative modification on the proinflammatory response of monocytic cell lines to trichomonad infection, confirming the importance of bacteria in modulating the inflammatory response [52].

The primary pathway of energy metabolism in *M. hominis* is the arginine dihydrolase pathway (ADI), which removes nitrogen from arginine and generates ATP. Margarita et al. reported that *M. hominis* also consumes extracellular arginine when in association with *T. vaginalis.* Depletion of arginine, facilitated by several microbial enzymes like arginase and arginine deiminase, is a common strategy employed by pathogens to evade the immune response [53]. In fact, human macrophages utilize free arginine to produce the toxic defense molecule nitric oxide (NO). *M. hominis* competes with macrophages for arginine in the host environment, reducing the amount available for NO production and thereby affecting their antimicrobial activity.

Altogether, these studies highlighted role of *M. hominis* in the modulation of the inflammatory process during *T. vaginalis* infection and led to the hypothesis of the possible impact in important pathologies associated with trichomoniasis, such as increased risk of HIV acquisition, and in cervical and prostate tumorigenesis [5,54,55].

## 4. *T. vaginalis* Symbionts: A Possible Role in Adverse Pregnancy Outcomes

Complications related to preterm birth (PTB) are responsible for about 27% of neonatal mortality. Intraamniotic infections and the induced uterine inflammation are among the main causes of PTB, and *Mollicutes* are the most frequently reported organisms in the amniotic cavity [56]. Vaginal *M. hominis* infection is associated with several adverse pregnancy outcomes and postpartum complications such as spontaneous abortion, stillbirth, preterm birth, low birth weight, and perinatal mortality [57]. The frequent detection of *M. hominis* in amniotic fluid and placental membranes of women with preterm pre-labor rupture of membranes (PPROM) seems to indicate a potential direct role of bacteria, triggering the synthesis of prostaglandins resulting in spontaneous preterm labor [58]. Interestingly, the same adverse pregnancy outcomes have been correlated with the presence of *T. vaginalis* that, differing from *M. hominis*, is unable to colonize the amniotic fluid [8,59,60]. The role of the protozoon was then restricted to the induction of a proinflammatory response, only indirectly leading to adverse pregnancy outcomes [49]. Despite the demonstrated association of trichomoniasis with adverse outcomes, the administration of metronidazole to eradicate the infection seems not to be able to prevent preterm delivery, but rather to increase the risk of PTB and low birth weight infants [61,62].

This apparently paradoxical effect may be explained by the high percentage of *T. vaginalis* strains harboring *M. hominis*: in this case, metronidazole treatment, effective on the protozoon and not on the symbiotic bacterium, could induce a massive release of *M. hominis* from killed trichomonad cells, that consequently are free to invade placental membranes and amniotic fluid. The demonstration that *M. hominis* released by metronidazole-treated *T. vaginalis* are able to infect WISH cells in vitro supports this hypothesis [63]. Ferrari de Aquino and Simoes-Barbosa recently represented, in a suggestive way, *T. vaginalis* as a piñata containing the different symbionts, which are released by the effect of metronidazole in vivo [64].

In *Mycoplasma hominis*, three genes (*alr*, *goiB* and *goiC*) have been found to be involved in invasion of the amniotic cavity [65]. Among the three genes, *goiC* is significantly associated preterm labor and can thus be considered a virulence trait of the *M. hominis* strains able to invade the amniotic cavity and the placenta [66]. The presence of the three genes has been investigated in *M. hominis* isolated from *T. vaginalis* strains, demonstrating a high percentage of positives (*alr* 96.55%, *goiB* 37.93%, and *goiC* 58.2%) [63].

Only a few studies so far have focused on possible correlations between *Ca.* M. girerdii and adverse pregnancy outcomes. Interestingly, Costello and colleagues detected 16S rRNA gene sequences of *Ca.* M. girerdii in the oral cavity of a low birth weight neonate, and both *T. vaginalis* and *Ca.* M. girerdii genomic DNAs were found in the saliva of a premature infant, suggesting vertical transmission during delivery [67,68].

## 5. *T. vaginalis* Metronidazole Susceptibility and the Symbiosis with Mycoplasmas

The standard treatment for trichomoniasis is based on nitroimidazole derivates: metronidazole (MTZ), which was only introduced in 1959; tinidazole; and secnidazole [69]. Although most *T. vaginalis* infections can be cleared using nitroimidazole drugs, an increasing number of drug-resistant *T. vaginalis* isolates has been reported in recent years [70,71,72].

The possible role of the symbiosis with *M. hominis* in the development of *T. vaginalis* resistance to metronidazole has been investigated by several groups, leading to controversial results. Xiao et al. observed a reduction in sensitivity to metronidazole in trichomonad isolates infected by *M. hominis*, while other groups reported a lack of correlation between the presence of the symbiont and drug resistance [46,73]. To shed more light on this debated issue, Margarita et al., in a recent study, investigated the possible correlation between the presence of symbionts and the in vitro drug susceptibility in *T. vaginalis* isolated in Italy and in Vietnam. The results of the study suggest that the presence of *M. hominis* is positively associated with a reduced resistance to metronidazole in *T. vaginalis*. The minimal lethal concentration of metronidazole was 2.9 µg/mL in Mh-positive and 5.9 µg/mL in Mh-negative strains (*p* < 0.05). Moreover, four out of five isolates showing a reduced sensitivity to MTZ were *Mycoplasma*-free [74]. Interestingly, this observation is consistent with results obtained by other research groups: in most cases, metronidazole-resistant strains observed in the other studies were *Mycoplasma*-free [35,36,75].

The role of *Ca.* M. girerdii on metronidazole resistance has been only recently investigated. In order to avoid any strain-to-strain variability often observed among clinical protozoan isolates, Margarita et al. generated a set of four isogenic *T. vaginalis*, differing only in the presence/absence of *M. hominis* and *Ca.* M. girerdii. Results demonstrated that *M. hominis* renders *T.vaginalis* more susceptible to MTZ, while the presence of *Ca.* M. girerdii seems not to influence the sensitivity of the protozoon [74].

Metronidazole resistance in *T. vaginalis* strain growth in aerobic conditions can be caused by conversion of toxic nitroradical form of antibiotic to the inactive prodrug due to high oxygen concentration inside the hydrogenosomes. Some authors have observed a decrement in the expression of hydrogenosomal proteins, including flavin reductase 1 (FR1), ferredoxin (Fdx) and pyruvate ferredoxin oxidoreductase (PFOR) in *T. vaginalis*-resistant strains [71,76]. The presence of *M. hominis* increased the expressions FR1 compared to the Mycoplasma-free *T. vaginalis* strain. This enzyme plays a crucial role in oxygen scavenging mechanisms leading to drug inactivation, so its upregulation in *T. vaginalis* when in symbiosis with *M. hominis* may explain, at least in part, their higher sensitivity to metronidazole [74].

## 6. Conclusions

The discovery and subsequent characterization of the intimate symbiotic relationship between *T. vaginalis* and *M. hominis*, both pathogenic to humans, open new perspectives for studying the complex interactions that pathogens establish not only with their human host but also among themselves.

The high incidence in the population of double infection by *T. vaginalis* and *M. hominis* suggests that the diagnosis must always consider the existence of both symbionts: the presence of a microorganism is highly suggestive of the simultaneous infection with the other. If both are not sought at the same time, in fact, the therapy will be directed only against a single symbiont, leaving the other free to continue to infect undisturbed. The recent discovery of the new symbiont of the protozoon *Ca.* M. girerdii makes the picture even more complex and strongly stimulates the study of the interactions that pathogenic symbionts can have not only with the host but also among each other and with the vaginal microbiota. In fact, in addition to the endosymbionts being able to influence virulence and immunopathogenesis of *T. vaginalis*, vaginal bacteria of dysbiotic microbiomes can also enhance the pathogenic capabilities of the parasite.

Therefore, the microbial consortium formed between the protozoan and its endosymbionts should not be regarded as a simple sum of microorganisms capable of causing separate infections in humans, nor should *Trichomonas vaginalis* be considered as a shuttle that passively transports bacteria. On the contrary, the in vivo presence of *T. vaginalis* in association with its symbionts and the interaction with the microbiota could represent a real consortium of pathogens with unique pathogenic characteristics to some extent different from those of single microorganisms.

## Figures and Tables

**Table 1:** List of studies showing the *Mycoplasma* spp. detection in *T. vaginalis* clinical isolates. *Ca.*Mg: *Ca.* M. girerdii; Mh: *M. hominis*.

Scheme	Geographic Origin	Detection Method	N. of Sample Analyzed	*Ca.*Mg +/ Mh − (%)	*Ca.*Mg −/ Mh + (%)	*Ca.*Mg +/ Mh + (%)
[33]	Italy	PCR	75	4/75 (5%)	21/75 (28%)	42/75 (56%)
[39]	China	PCR	6		4/6 (66.6%)	1/6 (16.6%)
